# The relationship between technology addictions and schizotypal traits: mediating roles of depression, anxiety, and stress

**DOI:** 10.1186/s12888-023-04563-9

**Published:** 2023-01-25

**Authors:** Feten Fekih-Romdhane, Haitham Jahrami, Rami Away, Khaled Trabelsi, Seithikurippu R. Pandi-Perumal, Mary V. Seeman, Souheil Hallit, Majda Cheour

**Affiliations:** 1 Faculty of Medicine of Tunis, Tunis Al Manar University, Tunis, Tunisia; 2 The Tunisian Center of Early Intervention in Psychosis, Department of Psychiatry Ibn Omrane, Razi Hospital, Tunis, Tunisia; 3 College of Medicine and Medical Sciences, Arabian Gulf University, Manama, Kingdom of Bahrain; 4 Ministry of Health, Manama, Kingdom of Bahrain; 5 High Institute of Sport and Physical Education of Sfax, University of Sfax, 3000 Sfax, Tunisia; 6 Somnogen Canada Inc, College Street, Toronto, Canada; 7 Saveetha Medical College and Hospitals, Saveetha Institute of Medical and Technical Sciences, Saveetha University, Chennai, India; 8 Department of Psychiatry, University of Toronto, Toronto, Canada; 9 School of Medicine and Medical Sciences, Holy Spirit University of Kaslik, P.O. Box 446, Jounieh, Lebanon; 10 Applied Science Research Center, Applied Science Private University, Amman, Jordan; 11 Research Department, Psychiatric Hospital of the Cross, Jal Eddib, Lebanon

**Keywords:** Schizotypy, Psychosis, Smartphone addiction, Internet addiction, Facebook addiction, Psychological distress

## Abstract

**Background:**

The way how technology addiction relates to psychosis remains inconclusive and uncertain. The present study aimed to test the hypothesis of a mediating role of depression, anxiety and stress in the association between three technology (behavioral) addictions (i.e., Addiction to the Internet, smartphones and Facebook) and psychosis proneness as estimated through schizotypal traits in emerging adults.

**Methods:**

A cross-sectional study was performed among non-clinical Tunisian university students (67.6% females, mean age of 21.5 ± 2.5 years) using a paper-and-pencil self-administered questionnaire.

**Results:**

Results for the Pearson correlation revealed that higher smartphone, Internet, and Facebook addictions’ scores were significantly and positively correlated with each of the depression, anxiety and stress subscores; whereas depression (r = 0.474), anxiety (r = 0.499) and stress (r = 0.461) scores were positively correlated with higher schizotypal traits. The results of the mediation analysis found a significant mediating effect for depressive, anxiety and stress symptoms on the cross-sectional relationship between each facet of the TA and schizotypal traits.

**Conclusion:**

Our findings preliminarily suggest that an addictive use of smartphones, Internet and Facebook may act as a stressor that exacerbates psychosis proneness directly or indirectly through distress. Although future longitudinal research is needed to determine causality, we draw attention to the possibility that treating psychological distress may constitute an effective target of interventions to prevent psychosis in adolescents with technology addictions.

## Introduction

The number of smartphone owners has been on a constant rise over the last six years, from 49% of the world’s population in 2016 to 83% in 2022 [1]. Smartphones provide easy, practical, and non-restricted access to many online services, with approximately three-quarters of Internet users accessing the Internet exclusively via smartphones by 2025 [2]. One of the most popular daily activities on the Internet is interaction with various social media platforms. These platforms include (but are not limited to) Facebook, YouTube, WhatsApp, Instagram, Tiktok, Twitter, Snapchat, the specifics depending on geographical region.

The amount of time Internet users spend on social media has also been increasing and has reached 144 min per day, an increase of more than 30 min/day since 2015 [3]. Such use has the potential of becoming rapidly addictive [4–7]; it has consequently been categorized as a new-era pandemic [8] and, in some countries, has become a public health problem [9]. In Tunisia, a substantial proportion of adolescents and young adults are reported at risk of internet [10–13], smartphone [14], and social media [15, 16] addiction.

Growing evidence supports the negative effects of the addictive use of digital technology on human physical and mental health. Being an addict to technology has repercussions on a youth’s abilities and habits, as well as on his/her social behavior [17]. It interferes with daily activities, school work, and academic performance [18–21]. Importantly, technology addiction (TA) negatively influences users’ emotional and social functioning [22–26], and correlates with the presence of psychopathologies such as depression, anxiety [22, 27, 28], insomnia [29], attention deficit hyperactivity disorder, and social anxiety disorder [4, 30]. However, although TA has been extensively investigated in relation to a wide range of mental health problems [15, 31, 32], there has been very little research thus far on how such addictions may influence the development of schizophrenia and related psychotic disorders [33–37]. Most of the literature on the relationship between TA and psychosis risk has been based on case reports documenting an emergence of psychosis during withdrawal from internet addiction (e.g., [38–40]).

### The relationship between TA and schizotypy

Schizotypy refers to a latent personality organization that manifests in several underlying subthreshold positive and negative psychotic symptoms, together with interpersonal difficulties [41]. Schizotypy is considered a potential precursor to formal schizophrenia-spectrum psychosis [42], thus offering a unique insight into the nature of the relationship between the behavioral TA and psychosis-proneness. A limited but increasing amount of studies suggests a potential link between TA and attenuated psychosis indicators among non-clinical youth, such as pre-psychotic symptoms [40, 43], brief psychotic episodes [16, 40, 44], high psychoticism scores [45], and schizotypal personality disorders [46, 47]. Recently, Massaro et al. [48] demonstrated a positive relationship between problematic technology use and schizotypal (mainly disorganized) trait levels among undergraduate students. Truzoli et al. [47] also highlighted associations between certain schizotypal traits (i.e., introverted anhedonia, impulsive nonconformity) and Internet addiction. Both schizotypy and TA share a myriad of characteristics, such as social anhedonia [49, 50], interpersonal deficits [32, 51, 52], impulsivity [53, 54], a propensity toward magical thinking [55, 56], and cognitive perceptual experiences [57, 58]. A recent study found that persisting patterns of problematic technology use was positively associated with an elevation of subthreshold psychotic symptoms over time, suggesting TA as “a new environmental stressor” contributing to the etiology of psychosis [33].

### Depression, anxiety, and stress as mediators between TA and schizotypy

There is increasing evidence that TA is positively related to self-perceived psychological distress [59–62]. At the same time, psychological distress has been found to be significantly linked to elevated psychosis risk, attenuated psychosis syndromes, and a heightened risk of transition from these to diagnosable psychosis [63, 64]. One hypothetical route from TA addiction to psychosis is the incursion of addiction into the time and energy needed to succeed in academic/professional tasks and interpersonal relationships. This, in turn, can lead to psychological distress and subsequent raise in psychosis risk [65]. Untreated psychopathology may add additional vulnerability to young adults with TA, and can be regarded as “a perpetuating risk of psychosis” [40]. To further explore the pathways between TA and psychosis, we hypothesized that psychological distress (i.e., depression, anxiety, stress) mediates the relationship between TA and schizotypal traits. A literature search revealed that scant research attention has been given to understanding the nature and the mediating factors in the relationship between TA specifically and psychosis proneness.

### Rationale and objectives of the present study

Exploring the interplay between behavioral addictions, psychological distress, and psychosis in young adults is relevant, given that this age group represents people at the peak of the onset of this psychopathology [66]. Additionally, today’s young adults are first exposed to technology at an early age and, thus, by adolescence, have been addicted for many years [67, 68]. Globally, nine out of ten youths report daily use of online activities [69], with smartphones and social media being preferred above all else [70–72]. Despite the available data, it is not known how TA can result in mental illness, psychosis in particular [73]. Investigating the mediating factors between TA and proneness to psychosis, as evidenced by the presence of schizotypal traits in adolescents may assist in designing effective intervention.

In this context, the present study aimed to test the hypothesis of a mediating role of psychological distress (depression and anxiety) in the association between three technology (behavioral) addictions (i.e., addiction to the Internet, smartphones, and Facebook) and the presence of schizotypal traits among non-clinical Tunisian university students. By researching different dimensions of TA (tech hardware, smartphone; tech software, Internet, and Facebook), we aim to provide a complete overview of how each dimension relates to schizotypy. In addition, by investigating all three psychological distress dimensions, we aim to offer a complete and thorough description of how each of these distress factors may serve as intermediaries between TA and schizotypy.

## Methods

### Participants and procedures

This is a cross-sectional study of Tunisian students enrolled at public universities in 2021–22. Eligibility criteria to participate were: age 18 or older, no personal history of psychosis, and no exposure to antipsychotic drugs. To be included, students needed to own a smartphone, have access to the Internet and a Facebook account. Participants were selected using convenience sampling over a three-month period (October-December 2021). A total of 745 students agreed to participate and provided informed written consent. After excluding incomplete questionnaires, 700 responses were used in the final analysis.

### Questionnaire

A paper-and-pencil self-administered questionnaire was used, containing two sections. The first section covered sociodemographic information, including age, gender, living arrangement, place of residence, and monthly family income. To evaluate the extent of participants’ smartphones, Internet, Facebook addictions, psychological distress, and schizotypal traits, the questionnaire included the following screening instruments in the second section:

### ***The Smartphone Addiction Scale – Short Version*** (SAS-SV, [74])

This is a 10-items research tool representing a shortened version of the original 33-items scale [75]. A total score is obtained by summing the items and ranges from 10 to 60. Higher scores point to an increased risk of smartphone addiction. The Arabic SAS-SV that we used in our study has good psychometric properties [76] and showed adequate internal reliability (Cronbach’s alpha = 0.88).

### ***The Internet Addiction Test*** (IAT, [77, 78])

The IAT is a 20-item measure used to evaluate dysfunctional Internet usage through probing feelings, productivity, social life, and sleeping pattern. The 5-point Likert scale yields total scores ranging from 20 to 100, with higher scores suggesting greater Internet addiction. The Arabic IAT employed in this study has good internal consistency [79] and revealed a Cronbach's alpha of 0.89 in the present study.

### ***The Bergen Facebook Addiction Scale*** (BFAS, [80])

The BFAS is a 5-point Likert-type 6-item scale, widely used to assess the six following features and symptoms of Facebook addiction: mood modification, conflict, salience, tolerance, withdrawal, and relapse [81]. Total scores may reach a maximum of 30, with higher scores indicating increased Facebook addiction. We utilized the Arabic version of the BFAS (Cronbach’s alpha = 0.87) [82], which has shown to reliability in our sample (alpha = 0.84).

### ***The Depression, Anxiety and Stress Scales*** (DASS-21, [83])

This consists of a 21-item measure used to assess the severity of psychological distress symptoms and is divided into three subscales: DASS-depression (7 items), DASS-anxiety (7 items), and DASS-stress (7 items). The DASS-21 is a four-point Likert-type scale (from “I strongly disagree” = 0 to “I totally agree” = 3), with higher scores in each subscale referring to greater distress. The Arabic DASS-21 [84] showed good reliability in this study, with Cronbach’s alpha for the total DASS-21 score of 0.93.

### ***The Schizotypal Personality Questionnaire*** (SPQ, [85])

The SPQ is a 74-item used to examine schizotypal traits and symptoms. It includes nine dimensions (i.e. Ideas of reference, paranoid ideation/suspiciousness, odd beliefs or magical thinking, unusual perceptual experiences, lack of close friends, excessive social anxiety, odd or eccentric behavior, odd speech, and constricted affect), divided into three subscales (i.e. positive, negative, disorganized). The higher the score, the more schizotypy features. The Arabic version of the SPQ [86] yielded good reliability with a Cronbach's alpha value of 0.91 for the total score (74 items).

### Statistical analysis

Data were analyzed using IBM SPSS 26.0 for windows (IBM Corp., Armonk, NY, USA). Shapiro–Wilk test revealed a normal distribution of data. Descriptive statistics were performed for the sociodemographic data. Then, the correlations between the SAS-SV, IAT, BFAS, DASS-21, and SPQ scores were evaluated using Pearson correlation coefficient analysis. Subsequently, mediation analyses were used to test the indirect effects of psychological distress in the relationship between each aspect of the TA (smartphone, Internet, and Facebook addictions) as independent variables and schizotypy as the dependent (outcome) variable. We conducted generalized linear models (GLM) equations for each distress dimension in predicting its mediation on the relationships between smartphone addiction-schizotypy, Internet addiction-schizotypy, and Facebook addiction-schizotypy, respectively. According to the models built, we hypothesized that: (1) The three independent variables (smartphone, Internet, and Facebook addictions) would affect the mediator variables (depression, anxiety, and stress) in the first equation, (2) smartphone, Internet and Facebook addictions would affect the outcome variable (schizotypy total scores) in the second equation; and (3) The three mediators (depression, anxiety, and stress scores) would affect schizotypy scores in the third equation. We also hypothesized that the effect of TA on schizotypal traits would be smaller in the third equation as compared to the second one.

## Results

### Participant characteristics

Participants were mostly females (67.6%), and had a mean age of 21.5 ± 2.5 years. More than half of the participants lived with their parents (57.4%), and the majority were from urban areas (87.3%). Further sociodemographic information is displayed in Table 1.

**Table 1:** Demographics and participants’ characteristics (*N* = 700)

	**Mean ± SD**	***N*** **(%)**
**Age**	21.5 ± 2.5 years	
**Gender**		
Male	227 (32.4%)	
Female	473 (67.6%)	
**Living arrangements**		
With parents		402 (57.4%)
With friends		260 (37.1%)
Alone		38 (5.4%)
**Monthly family income**		
< 500 TD		49 (7.0%)
500 – 1000 TD		186 (26.6%)
1000 – 2000 TD		244 (34.9%)
2000 – 3000 TD		123 (17.6%)
> 3000 TD		98 (14.0%)
**Residency**		
Urban area		611 (87.3%)
Rural area		89 (12.7%)

### Pearson correlation coefficient analysis

Results for the Pearson correlation between study variables are presented in Table 2. Higher smartphone, Internet, and Facebook addictions’ scores were significantly and positively correlated with each of the depression, anxiety and stress subscores; and depression (r = 0.474), anxiety (r = 0.499) and stress (r = 0.461) scores were positively correlated with higher schizotypal traits.

**Table 2:** Correlation matrix (Pearson's r) between the study variables

	IAT scores	BFAS scores	SAS-SV scores	DASS-Depression	DASS-Anxiety	DASS-Stress
IAT scores	1					
BFAS scores	0.560^**^	1				
SAS-SV scores	0.439^**^	0.382^**^	1			
DASS-Depression	0.379^**^	0.280^**^	0.226^**^	1		
DASS-Anxiety	0.375^**^	0.307^**^	0.234^**^	0.733^**^	1	
DASS-Stress	0.382^**^	0.277^**^	0.317^**^	0.785^**^	0.787^**^	1
SPQ scores	0.277^**^	0.238^**^	0.186^**^	0.474^**^	0.499^**^	0.461^**^

^**^ p < *0.001*

*IAT  Internet Addiction Test, BFAS  Bergen Facebook Addiction Scale, SAS-SV  Smartphone Addiction Scale – Short Version, DASS-21  Depression, Anxiety and Stress Scales, SPQ  Schizotypal Personality Questionnaire*

### Mediation analyses: direct and indirect associations of TA with schizotypy

**Fig. 1:**
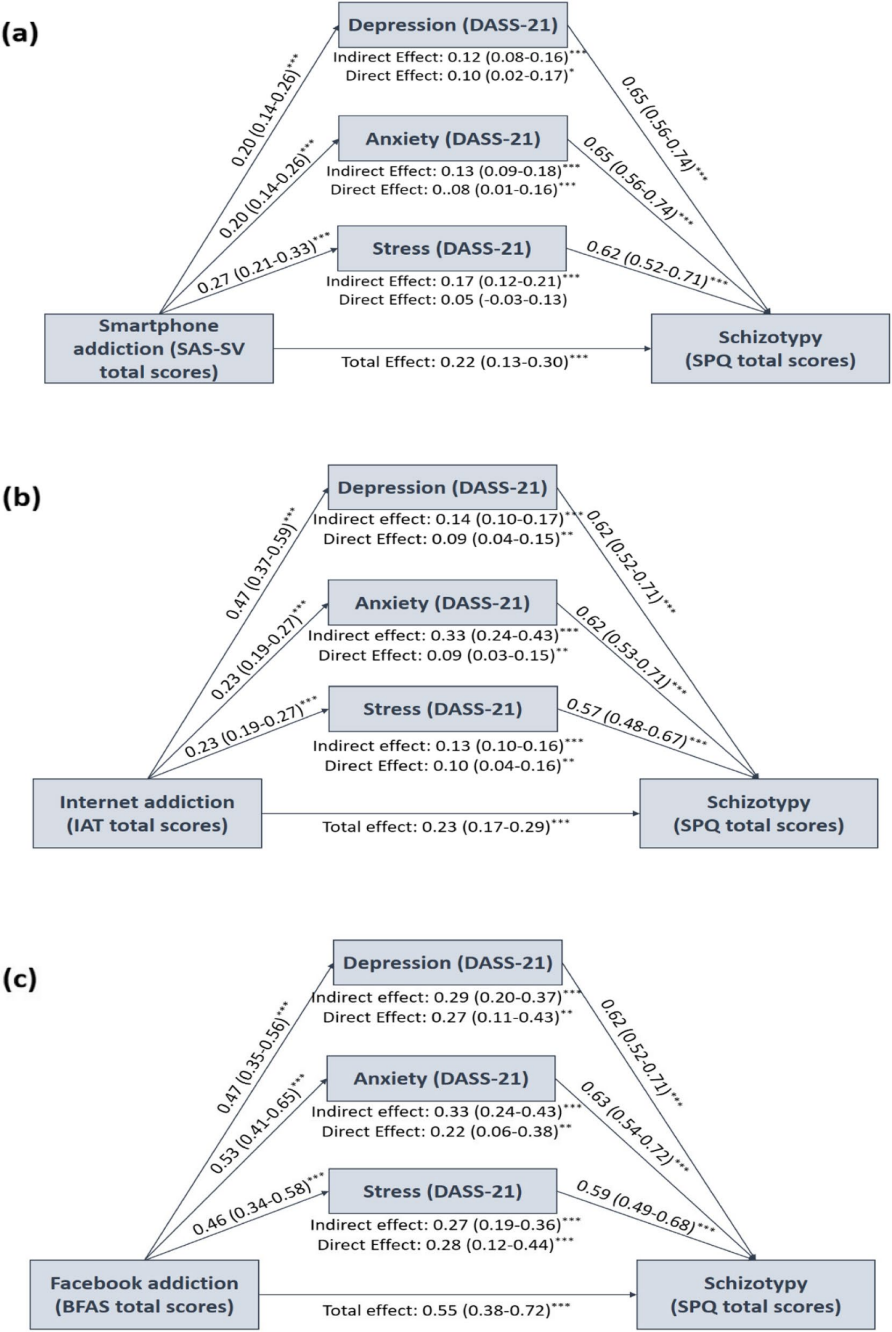
The estimation of the direct and indirect effects of depression, anxiety and stress between TA and schizotypy. Each line is labeled with effects estimate and their 95%- confidence intervals. IAT: Internet Addiction Test; BFAS: Bergen Facebook Addiction Scale; SAS-SV: Smartphone Addiction Scale –Short Version; DASS-21: Depression, Anxiety and Stress Scales; SPQ: Schizotypal Personality Questionnaire. ^*^p < 0.05, ^**^p < 0.01, ^***^p < 0.001

The results of the mediation analysis found a significant mediating effect for depressive, anxiety and stress symptoms on the relationship between each facet of the TA and schizotypal traits (Fig. 1).

## Discussion

This work focused on elucidating cross-sectional relationships between TA and schizotypy in samples of non-clinical emerging adults and deepening knowledge about the nature of the interaction between these variables on psychological distress (the mediator variable). The main finding was that, as expected, the mediation paths revealed that depression, anxiety, and stress played a significant indirect role in the association between each TA facet investigated and schizotypal traits.

Findings showed that the relationships between each independent variable (i.e., smartphone, Internet and Facebook addictions) and the dependent variable (i.e., schizotypy) were significant. These results are in agreement with previous literature that showed that excessive digital technology use relates to a wide range of psychopathology symptoms and manifestations, including attenuated psychotic symptoms [16, 40, 43, 44] and schizotypal personality traits and disorders [46–48]. TA has even been suggested by some authors as an environmental risk factor that interacts with genetic vulnerability to “cause” psychosis [33, 38–40]. Indeed, the recent literature has documented a link between Internet addiction and substantial structural brain changes [87] in regions closely associated with subclinical psychotic symptoms [88]. This might suggest that changes in a developing brain made vulnerable by TA confer an elevated risk of psychosis development by harming analogous brain pathways. Furthermore, a genetic link between TA and psychosis is also possible. A specific polymorphism in the CHRNA4 gene (gene coding for the nicotinic acetylcholine receptor subunit alpha 4, rs1044396) has been identified in individuals presenting with internet addiction [89], and a similar profile of genetic polymorphisms has been found in schizophrenia [90]. In addition, the serotonin genotype 5-HTTLPR (short alleles of the serotonin transporter gene promoter region) has been identified in Internet addiction [91] and schizophrenia [92]. Other explaining mechanisms of the relationship between TA and psychosis can be hypothesized, including negative cognitions about the self and world (e.g., beliefs that one is more valuable online than offline or that the real world is unsafe compared to the world online), which have been reported in both TA [93, 94] and psychosis [95, 96]. We are aware, however, that although we have demonstrated a significant cross-sectional relationship between TA and schizotypy, longitudinal studies are still needed before any causal conclusions can be drawn. It is plausible that the association between these two variables is bidirectional, and that highly schizotypal individuals are more prone to show behavioral addictions in comparison to healthy people. Indeed, people with high schizotypy have certain traits that may increase their vulnerability to develop TA, such as excessive social anxiety [97], having poor social support and no close friends [98]. We thus urge readers to interpret our results with caution.

Overall, these data suggest that the interaction between TA and psychosis is rather complex, and likely underpinned by several factors and mechanisms. Hence the importance of further exploring the pathways linking TA to psychosis and psychosis vulnerability by considering potential mediators. To our knowledge, our study is the first to show that depression, anxiety and stress can act as mediators in the cross-sectional association between TA and schizotypy among non-clinical emerging adults. Our findings are consistent with the evidence of the previously established positive association between TA and psychological distress on the one hand [59–62, 99, 100], and between distress and schizotypy symptoms on the other [101, 102]. These data, along with our findings, suggest that the relationship between TA and schizotypy is not only direct but is also mediated by the action of psychological distress. Therefore, the presence of depression, anxiety and stress, either as a result of excessive digital technology use or as pre-existing conditions [103], partially explains the relationship between TA and schizotypy. In other words, the various forms of TA examined in this study might have been associated with more severe symptoms of schizotypy through symptoms of depression, anxiety and stress.

However, again, we are aware that our findings should be interpreted cautiously because of the study’s limitations. The cross-sectional research design of our study can say nothing definitive about causality. Future prospective longitudinal research studies are needed. Secondly, despite the use of psychometrically valid and reliable measures, the self-report nature of our survey questions its accuracy. Thirdly, while three aspects of TA, namely smartphones, the Internet, and Facebook, have been considered in this study, the latter aspect was assessed only in reference to the Facebook platform. Participants who mainly have used other platforms (e.g., Instagram, YouTube) and Facebook non-users were omitted from this study, limiting its representativeness of Tunisian youth. Participants were mainly women; it is possible that the results may not speak as accurately for men. Youth from other cultures and geographical regions may also not be represented.

### Study implications

Although preliminary, the present research shows that all three facets of TA are strongly and positively associated with schizotypy among non-clinical emerging adults. It is hoped that these results will help parents, educational institutions, clinicians, and researchers understand the effects of TA on young individuals vulnerable to psychosis. Previous longitudinal research [33] found that continued problematic technology use was associated with persistence and worsening of psychotic symptoms and experiences, whereas discontinuation of usage was followed by a significant improvement in symptoms. This only preliminarily supports previous conclusions that technology addiction may, like cannabis use at this time of life, serve as an environmental stressor that “unmasks the subtle vulnerability” to psychosis [33] as per the diathesis-stress model of psychosis [104]. Previous data combined with our findings draw attention to the possibility, and likely benefits, of considering TA as a target for prevention and early intervention in psychosis. Until now, the development and implementation of prevention and intervention strategies for technology-related addictions have been addressed poorly in empirical research. A few psychosocial interventions, such as cognitive-behavioral therapy (CBT), motivational enhancement therapy [105], and mindfulness techniques [106] have shown positive results for TA, but these and other psychotherapies need more serious study.

This study provides initial evidence that depression, anxiety, and stress may serve as strong mediators in the relationship between TA and schizotypal traits. Consequently, treating psychological distress may constitute an effective target for intervention and prevention of psychosis in adolescents with TA. Technology-based interventions for psychosis prevention (e.g., web-based psycho-education, integrated web-based therapy, web-based CBT, text messaging interventions, social networking, and peer and expert moderation) [107] may be highly acceptable, relevant, and feasible form of reaching youth.

## Conclusion

By testing the mediating paths between TA and schizotypy, this study provides data that have not been researched before. The findings of this study in Tunisian university undergraduates has revealed a significant mediating role of depression, anxiety and stress in the cross-sectional relationship between TA and schizotypy. This preliminarily supports prior assumptions that the addictive use of smartphones, the Internet, and Facebook at vulnerable ages, when brain circuits are still being developed, may act as a stressor that directly or via psychological distress, can increases the risk for psychosis. This deserves the attention of parents, educators, counselors, and clinicians working in early intervention services. Finally, although the current findings are suggestive, they must be considered preliminary until further research is able to longitudinally replicate the association between TA and schizotypy.

## Data Availability

All data generated or analyzed during this study are not publicly available due to the privacy of the participants' identities. The dataset supporting the conclusions is available upon request to the corresponding author.
